# Toward Multidimensional Front-of-Pack Labels: Integrating Nutritional, Environmental, and Processing Information

**DOI:** 10.3390/nu17142258

**Published:** 2025-07-08

**Authors:** Luca Muzzioli, Lucia Maddaloni, Maria Pintavalle, Eleonora Poggiogalle, Olivia Di Vincenzo, Silvia Migliaccio, Giuliana Vinci, Lorenzo Maria Donini

**Affiliations:** 1 Department of Experimental Medicine, Sapienza University, 00185 Rome, Italy; maria.pintavalle@uniroma1.it (M.P.); eleonora.poggiogalle@uniroma1.it (E.P.); olivia.divincenzo@uniroma1.it (O.D.V.); silvia.migliaccio@uniroma1.it (S.M.); lorenzomaria.donini@uniroma1.it (L.M.D.); 2 Department of Management, Sapienza University, 00185 Rome, Italy; giuliana.vinci@uniroma1.it

**Keywords:** front-of-pack labelling, FOPL, nutrition, sustainability, food processing, consumer behaviour, NOVA classification, eco-score, Nutri-Score

## Abstract

Front-of-pack labels (FOPLs) have been identified as a potential key tool to enable consumers to make healthier and more sustainable food choices. The simplification of complex nutritional, environmental, and processing data into clear and immediate formats is an essential function of FOPLs, which facilitates a more efficient connection between detailed product information and real-world purchasing decisions. This review critically evaluates the three main categories of FOPL—nutritional (e.g., Nutri-Score), environmental (e.g., Eco-Score) and processing-based (e.g., NOVA)—and examines emerging efforts to weave these dimensions into unified labelling frameworks. A bibliometric analysis of 1803 publications from Scopus, Web of Science, and Google Scholar was conducted, using VOS viewer to identify co-occurrence networks and thematic clusters. A narrative synthesis of label design methods, regulatory steps and consumer impact research followed this. Despite the considerable maturation of individual FOPLs, their combined application remains ad hoc. Establishing harmonized, multidimensional criteria is therefore essential to ensure consistent labelling that informs consumers and promotes public health and sustainability goals.

## 1. Introduction

In recent decades, the function of food labels has undergone a significant transformation, evolving from a tool for identification to a communication key for consumers. The concept of front-of-pack labels (FOPLs), which are designed to convey crucial information about food products concisely and immediately, is an integral component of this. The FOPLs, which are placed on the front of packs, adopt a variety of formats, ranging from ‘Traffic Light’ colour systems to alphabetical scales (e.g., Nutri-Score) [1], graphic symbols to numerical judgements (e.g., Eco-Score, Carbon Trust Label) [2,3]. This visual simplification is a response to the contemporary consumer’s need to swiftly evaluate the nutritional profile of food items, encompassing their sugar, saturated fat, salt, and fibre content. It also addresses the consumer’s desire to ascertain the environmental impact of food and the extent of its industrial processing.

Three main categories of FOPLs are commonly distinguished: (i) nutritional FOPLs summarize the overall nutritional quality, translating analytical parameters into comparable scores across products [4]; (ii) environmental FOPLs quantify the ecological footprint, measuring factors such as greenhouse gas emissions, water consumption, and land use throughout the life cycle [5]; and (iii) food processing labels, finally, classify the degree of transformation, offering indications of the level of industrial manipulation undergone by foods [6]. In addition to these stand-alone systems, hybrid approaches are emerging that integrate multiple information criteria into a single graphical solution, thereby providing a holistic and multidimensional view of the product [7].

Globally, food labelling has become a key public health instrument to inform consumers and encourage healthier choices [8]. In the context of nutrition policy, countries such as Chile, Mexico, and Israel have implemented mandatory FOPL systems, which use colour-coded labels to indicate the sugar, salt, or fat content of food items. The efficacy of these labelling systems in influencing consumer behaviour and food reformulation has been a subject of considerable research [9,10]. These efforts have designated a more extensive international initiative to promote transparent and accessible nutritional information. In Europe, a significant development was the implementation of Regulation (EU) No 1169/2011, which stipulated the mandatory display of key data elements, including the product name, the complete ingredient list, allergens, and nutritional values [11]. While this table-based format is essential for public health and transparency, it is often difficult to interpret quickly while shopping [11]. The European Union is currently discussing the introduction of new nutrition labels on packages. As part of a common strategy, it is planned to define a standard front-of-pack format within a set deadline [12,13]. The studies are underway to assess the influence of these systems on consumer decisions and perceptions of health and sustainability. These global initiatives are indicative of a more extensive movement towards enhancing transparency and supporting healthier food environments through the utilization of simple, accessible visual indicators.

The objective of this review is to undertake a critical and discursive examination of front-of-pack (FOPL) nutrition, environmental, and processing labels, with a particular focus on label design and methodologies employed in a multidimensional model.

## 2. Materials and Methods

Two different approaches were used to provide an overview of FOPLs. Firstly, a bibliometric network analysis was conducted to analyze the current trends in the field of FOPLs. Then, a literature review was conducted to understand more in detail the different dimensions investigated by FOP research.

### 2.1. Network Analysis

The bibliometric analysis was conducted to identify links between bibliometric networks. The VoS viewer software (Ver. 1.6.20) (http://www.vosviewer.com/ accessed on 1 February 2025) was utilized to construct a network map by clustering both keywords and descriptive attributes extracted by selected studies [14]. VoS clustering methodology was applied to search results in the Scopus databases, obtained by applying the search string (“front-of-pack*” OR “front of pack*” OR “FOP”). The initial search yielded 1224 articles, which were then harmonized by removing duplicates and normalizing authors, journals, and publications. Network analysis, specifically co-authorship analysis, was conducted to identify which states were most actively involved in publishing articles on this topic. The subsequent stage of the research involved conducting an analysis of keyword co-occurrence related to the authors’ keywords.

### 2.2. Literature Review

Four distinct searches of the Scopus, PubMed, and Google Scholar databases were conducted to identify publications belonging to the different dimensions of FOPLs: nutritional, environmental, food processing, and studies combining multiple dimensions. For every search, publications were selected after removing duplicates and normalizing author, journal, and publication period information, thus ensuring data consistency.

The strings associated with the four areas are as follows:

(i) Nutritional FOPLs: [FOP AND (nutritional label OR Nutri-Score OR Food nutrition OR food choice), [food AND (nutritional AND nutrients AND health choice) AND (label OR labelling)];
(ii) Environmental FOPLs: [FOP AND (ecolabel OR ecolabeling)], [food AND (sustainability OR sustainable OR environmental) AND (labelling OR label)];
(iii) Processing FOPLs: (Food processing OR Processed food OR Food classification OR Ultra-processed food OR processed food OR Food processing classification) AND (classification system OR classification OR system);
(iv) Combined FOPLs: FOP AND (ecol* OR eco-* OR sustainab* OR enviro*) AND (food processing OR NOVA OR UPF); FOP AND (ecol* OR eco-* OR sustainab* OR enviro*) and FOP AND nutrition AND (food processing OR NOVA OR UPF).

Front-of-pack labelling is a relatively recent field of research; therefore, the literature searches were not filtered by year or language of publication. For the same reason, all article categories were included, provided that the paper was available as full text.

## 3. Results

### 3.1. Bibliometric Analysis

**Figure 1:**
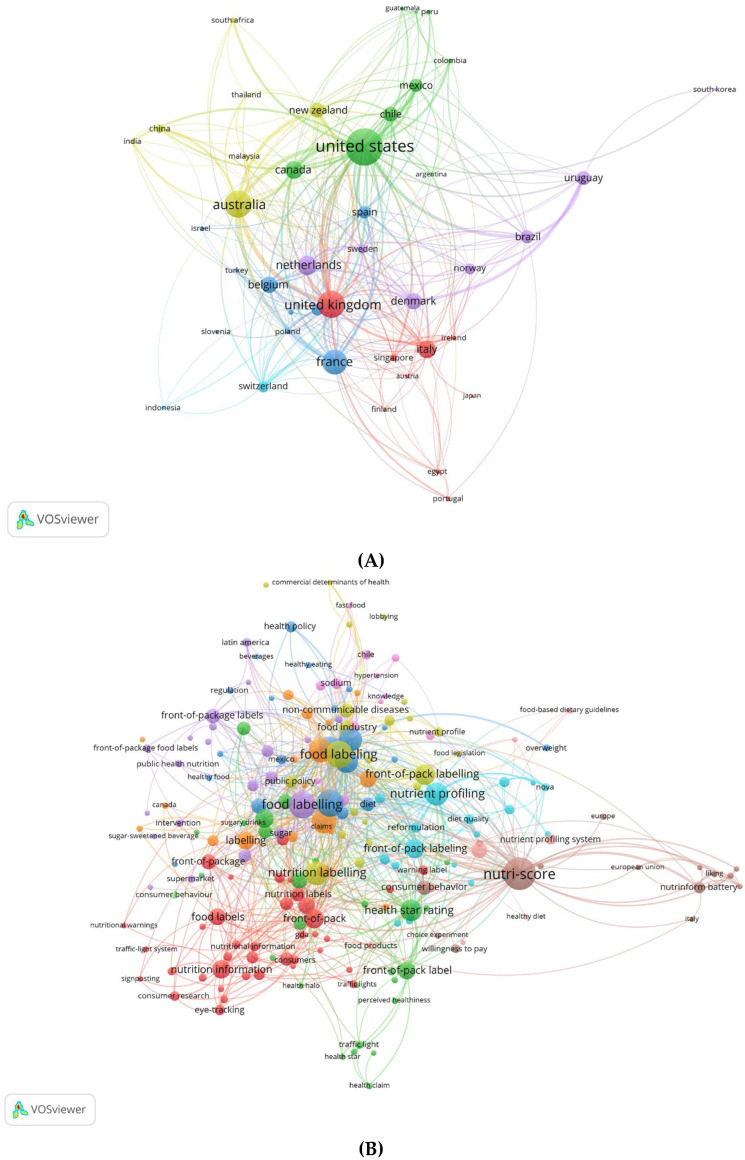
The bibliometric network of the literature on front-of-pack labelling: (**A**) co-authorship analysis of countries (6 clustering); (**B**) co-occurrence analysis of authors’ keywords (11 clustering). The clusters relate to the main research topics, highlighting the significant role of front-of-pack labelling in nutrition policymaking, consumer information communication, and food industry regulation.

A bibliometric approach was adopted to map the research landscape on front-of-pack labelling, focusing on food aspects. The primary bibliometric analysis, conducted with the VOSviewer software (Ver. 1.6.20), performed a co-authorship analysis of countries in order to investigate those in which there is a greater interest in the field of FOPLs. As observed in Figure 1, the United States has shown the greatest activity in this research field, followed by Australia, the United Kingdom, France, Italy, and Belgium. The highest number of publications has been particularly related to the development of early nutritional FOPLs (for example, Nutri-Score, Health Star Rating, Warning Labels, etc.). Recent developments have been observed in several other states, including Brazil, Mexico, and Chile, that have demonstrated a specific interest in the food-processing dimension of food labelling.

On the other hand, the map displayed in Figure 1B shows the thematic structure of the scientific literature on FOPLs. This is realized through a clustering algorithm based on modular optimization, where terms are grouped into conceptually related sets, identified by specific colours. A significant and concentrated cluster (blue) has formed at the core of the map, centred on the terms “food labelling” and “front-of-pack labelling.” This cluster represents the core of the literature, where general discussions of food labelling, definitions and regulatory implications are concentrated. Additional thematic clusters emerge around this core. The red cluster focuses on consumer aspects such as “nutrition information” and “eye-tracking”—how labels influence perception and purchasing. The green cluster emphasizes the perceived impact of labelling, using terms like “willingness to pay” and “perceived healthiness”. Other thematic clusters focus on specific areas: the purple cluster on public health and health policies, and the yellow cluster on economic pressures. The brown cluster indicates connections between “Nutri-Score”, “nutrient profiling”, “European Union”, “NutrInform Battery”, and “Italy”. This cluster reflects the policy relevance and European debate on nutrition labelling systems. The map shows the interdisciplinary nature of nutrition labelling research, although it lacks thematic clusters on environmental labels, food processing labels, or a combination of these. This highlights that, despite their importance in the current debate on nutrition labelling, they are not yet central to the scientific literature on the topic.

### 3.2. Literature Review

**Figure 2:**
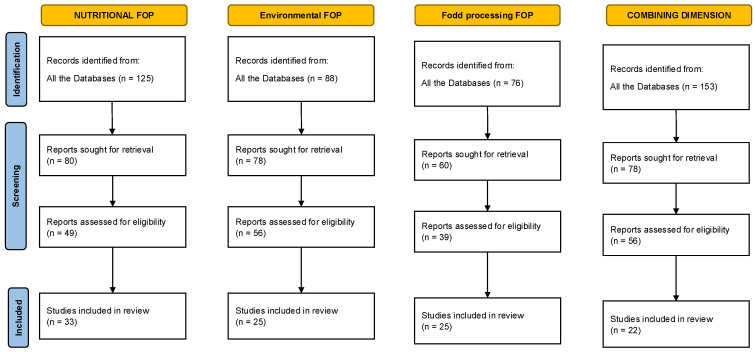
Flow diagram of literature search. Searches were conducted separately for each dimension: nutritional, environmental, and food processing labels; a further search focused on labels that combined multiple dimensions.

The results of the review are presented in Figure 2, which illustrates the process of item selection and distribution for each of the four dimensions analyzed. The figure follows a scheme inspired by the PRISMA model, adapted to reflect the hybrid and thematic nature of the methodological approach, which combines a systematic selection with an exploratory bibliometric analysis divided into the four dimensions of FOPLs.

#### 3.2.1. Front-of-Pack Nutritional Label

**Figure 3:**
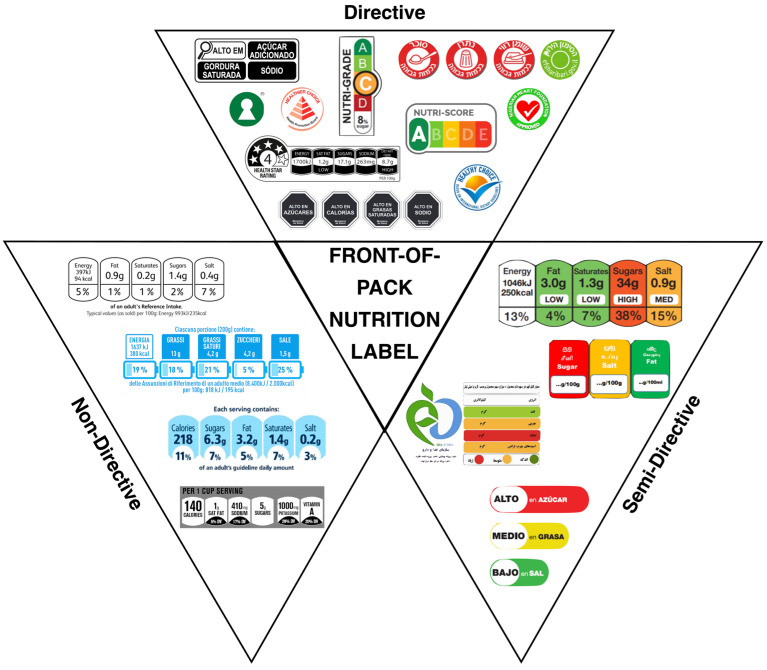
Classification of front-of-pack nutritional labels. They are divided into three groups: directive, semi-directive, and non-directive labels depending on whether they provide, respectively, a summary indication of healthiness, a healthiness indication per single nutrient, or nutrient amounts in the food product.

In the last decade, there has been an increasing use of front-of-pack nutrition labels. These labels, integrated with the traditional back-of-pack (BOP) information, aim to educate consumers and stimulate the food industry to reformulate their products to improve their nutritional profile and, consequently, their impact on public health [15,16]. Therefore, nutrition labels aim to inform consumers about the nutritional value of packaged foods and their contribution to the overall dietary composition. These labels arise from the need to offer consumers a valid tool to promote healthy and nutritionally balanced diets to prevent obesity and diet-related non-communicable diseases. This type of labelling was introduced in the 1980s and, to date, they are adopted, either mandatorily or voluntarily, by numerous states in different graphic and communication formats [17]. Mandatory labels, such as warning labels adopted by Chile, must be displayed on food packaging, unlike voluntary labels, e.g., the Health Star Rating adopted by Australia and New Zealand, for which manufacturers can autonomously choose whether to display them. Figure 3 shows front-of-pack nutrition labels classified by typology.

Nutritional FOPLs can be classified into informative labels (e.g., NutrInform Battery, Reference Intakes, etc.) and interpretive labels (e.g., Nutri-Score, Warning labels, etc.). The former provides easy-to-understand information regarding the nutrients contained in the food by highlighting them using numerical information (non-directive labels) or combined with colours and symbols (semi-directive labels) [17,18]. This type of label, which is widely used in the United States, Latin America, and the European Union, has five main symbols that refer to the energy (KJ or Kcal) and nutrient (g) content per serving, specifically the content of calories, total and saturated fat, sodium, and sugars, which represent the main daily nutrients [19]. Some of these labels, such as the NutrInform Battery, the Reference Intakes label, Facts Up Front, the UK Front Of Pack label and the Traffick Light Labels, also report the percentages per serving of the reference daily intake [8,19,20,21]. Some of these labels, such as the Traffick light label and the UK Front Of Pack label, are also associated with colours ranging from red to green concerning the concentration of nutrients in the food in question [8,17,19].

Another type of label is the evaluative label, also called interpretative or directive, which provides an overall judgement of the nutritional quality of the product considering different nutrients in the food, such as saturated fatty acids, simple sugars, vitamins, minerals, etc. [4,15,18]. The latter can be further subdivided into “positive judgement labelling”, “warning labelling”, and “classification or spectrum labelling” [21,22]. The first type of labelling, known as “judgement logos”, is designed to provide consumers with concise and comprehensive information on the nutritional quality of a food in a group of homogeneous foods. This system is based on the aggregation of nutritional information on the label, which is combined to produce a quality score or rating. In other words, information on energy, fat, sugar, salt, and other nutrients is aggregated and transformed into an indicator that directly expresses whether the nutrient content of the food is more or less favourable for health. Such a rating logo (e.g., Keyhole or Healthy Choice logo) allows the consumer to compare different products quickly and easily, thus facilitating more informed food choices [22].

Warning labels are communication tools designed to highlight the potentially harmful nutritional characteristics of foods clearly and immediately, to guide consumers to make healthier choices. These labelling systems use recognizable logos and symbols to highlight the presence of excess components such as sugar, saturated fat, or sodium. This type of labelling, which is widely used in South America, was developed to provide transparent and easily understandable information directly on the food package [20]. The aim is not to judge the food as ‘good’ or ‘bad’, but rather to immediately alert the user to potential health risks associated with excessive consumption of certain nutrients. In this way, warnings help consumers to make informed choices, promote a more balanced diet, and contribute to the prevention of diet-related diseases. In addition, introducing these labels has a positive impact on the manufacturing sector by encouraging manufacturers to reformulate their recipes to reduce the content of harmful ingredients and improve the overall nutritional profile of their products. Thus, in addition to guiding individual choices, health warnings play a key role in promoting a healthier approach to food at a collective level [15,19].

The third type of interpretative labelling is ‘grading or spectrum labelling’; this type of labelling aims to inform consumers about the nutritional quality of food products by considering all nutritional parameters that affect quality. Thus, not only nutrients potentially harmful to health, such as salt or unsaturated fatty acids, are considered, but also the content of other nutrients such as vitamins, minerals, fibres, etc. [21,23]. This type of labelling includes the Nutri-Score, which was born in 2017 in France and implemented in 2023 and is now adopted in several European countries [13,24,25,26]. This type of labelling summarizes the nutritional quality of food in a five-level graphical scale (A to E) to which colour (from dark green to red) is associated, complementing the mandatory information of Regulation (EU) No 1169/2011 [11,13,27]. It is based on an algorithm that evaluates the contents of favourable and unfavourable nutrients for health and transforms it into a nutritional score [28]. Interpretative labels also include the Health Star Rating Label, which was developed in Australia and New Zealand to assess the overall nutritional quality of foods simply and visually. It assigns a score expressed in stars from 0.5 to 5, with a higher number of stars indicating a more favourable nutritional profile. The system is based on a scientifically validated algorithm that considers nutrients to watch—such as energy, saturated fat, total sugars, and sodium—as well as beneficial nutrients such as fibre, protein, and the amount of fruit, vegetables, and nuts. In this way, the Health Star Rating helps consumers quickly compare products and make more informed food choices, promoting healthier diets [22,29,30,31].

Front-of-pack nutrition labels offer several advantages. First, they simplify and make complex information immediately accessible, facilitating comparisons between products and helping consumers make more informed food choices. They can also incentivise manufacturers to improve the nutritional quality of their products and support public health policies by contributing to the prevention of diet-related diseases. However, they also have some disadvantages [13,23,32]. The simplification required to make information immediately understandable can sometimes overlook important details or create overly reductive interpretations of nutritional quality. Furthermore, the variety of systems in use and the complexity of the calculation algorithms may lead to confusion among consumers, especially if the evaluation criteria are not always transparent. Finally, there is a risk that such labels will be perceived as marketing tools rather than true health indicators if not accompanied by adequate information campaigns [16,33,34].

#### 3.2.2. Environmental Food Label

In recent years, alongside the development of nutrition labels, there has been a tendency towards FOPLs that provide information regarding the environmental impact of food products [35,36]. These labels, which have been developed in response to the growing demand for transparency and sustainability, are designed under a range of regulations and methodological standards, including ISO 14024:2018 [37] and 14021:2016 [38] for communication and ISO 14040:2021 [39] and 14044:2021 [40] for environmental impact assessment [5,41]. Despite the absence of specific European regulations, the ISO 14000 standard family offer a framework for the design, verification, and communication of environmental performance [42]. The European Union is currently engaged in efforts to harmonize FOPL environmental labelling by promoting the adoption of methodologies based on the Environmental Footprint (EF). In this context, the Product Environmental Footprint (PEF) and the Organization Environmental Footprint (OEF) represent two key tools: PEF offers a standardized method for calculating the environmental impact of a product over its entire life cycle, while OEF applies a similar approach at the organizational level. These methodologies ensure a reliable, consistent, and comparable framework for the quantification of environmental impacts, thus promoting transparency and accessibility of data [43]. A central aspect in the development of these labels is the need to counteract greenwashing, i.e., the dissemination of misleading or unverified information on environmental sustainability. In this regard, the EU has initiated regulatory measures, including Directive 2024/825/EU and the Green Claims Directive [35,44].

The primary objectives of these measures are threefold: firstly, to standardize methodologies for calculating environmental impacts; secondly, to ensure that claims are supported by independently verifiable data; and thirdly, to regulate the use of claims such as ‘low carbon’ or ‘sustainable product’. A variety of FOPL labelling systems are currently employed throughout Europe to facilitate effective communication of the environmental impact of food products. These labels diverge in their calculation methods (single or multi-impact), the indicators studied (e.g., carbon footprint, water footprint, etc.), the type of scoring (colour or letter scales), and the graphic elements that facilitate the interpretation of the information by the consumer [45].

Table 1 provides a comprehensive sample of these labels, covering methodologies, indicators, scoring systems, and graphical elements. The environmental labels examined—Eco-Score, Planet-Score^®^, Carbon Trust Label, Foundation Earth Eco-Impact Label and Enviroscore—are key tools for communicating the environmental impact of food products in a transparent and accessible way. While sharing the common goal of supporting consumers and producers in making sustainable choices, each adopts specific methodologies and approaches, highlighting environmental aspects and offering different communication formats.

It is noteworthy that most of these systems are founded on well-established scientific methodologies, including Life Cycle Assessment (LCA) and Product Environmental Footprint (PEF). The utilization of LCA, as delineated by ISO 14040:2021 [39], facilitates the evaluation of the environmental impact across the entire product life cycle, encompassing the collection of raw materials through to the final disposal stage [2,35]. This approach is common in both the Eco-Score and the Foundation Earth Eco-Impact Label and Enviroscore, although each system supplements it with additional indicators to highlight specific aspects, such as water consumption, greenhouse gas emissions, biodiversity, pesticide use, and animal welfare [27,35,36].

**Table 1:** The main environmental front-of-pack labels and environmental impact methodology applied.

Name	State	Methodology	Score	Infographic	Refs.
**Eco-Score**	France	LCA + PEF	From A to E		[2,35,41]
**Planet-Score**	France	LCA + PEF	Icons + numeric		[25,42]
**Carbon Trust Label**	UK	Carbon FootPrint	Numeric	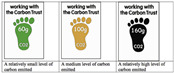	[3,43]
**Eco-Impact label**	UK	LCA	From A^+^ to G	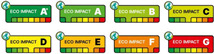	[35,44]
**Enviroscore**	France	LCA + PEF	From A to E		[45,46]

The Eco-Score, for instance, is distinguished by the application of a bonus–malus system that integrates five additional indicators—production methodology, short supply chain, environmental commitment, packaging sustainability, and impact on biodiversity—that can modify the basic score [2,41]. The result is expressed on a numerical scale from 0 to 100 and divided into five categories, where category A indicates the lowest environmental impact and category E the highest [2,27]. The Planet-Score^®^ was created in response to the limitations of the traditional PEF, updating some indicators and integrating aspects such as biodiversity loss, pesticide use, animal welfare, and local ecosystem overload [25,42]. Based on the principles of Life Cycle Thinking (LCT), this system provides the consumer with a score like the Eco-Score, expressed on a scale from A to E, but enriched with specific information on key environmental impacts [36,47].

In contrast with the other environmental label, the Carbon Trust label has been developed with a focus on the carbon footprint, measured in kilograms of CO_2_ equivalent per unit of product [43,48]. The label is based on international standards, such as PAS 2050, and analyses all stages of the product life cycle, from production to distribution and disposal, providing an accurate quantitative assessment of greenhouse gas emissions (carbon footprint) [43,48]. The Foundation Earth Eco-Impact Label, launched in the UK, employs a comparable approach by integrating LCA and PEF. However, it differs in its emphasis on agricultural practices and pesticide use, as well as deforestation [35,44]. The final score expressed on a scale from A+ (very low impact) to G (very high impact) is supported by an intuitive visual system where colour (from green to red) immediately reinforces the message about the level of sustainability of the product. Finally, the Enviroscore represents an endeavour to harmonize the assessment of the environmental impact of food products, once more based on the PEF. The data is normalized against reference standards such as the ‘European Food Basket’, which encompasses 23 categories representative of European consumption [45,49]. The environmental impacts are then aggregated into a single index—the European Food Environmental Footprint Single Index (EFEFI)—and translated into a score on a five-point scale from A (‘very low impact’) to E (‘very high impact’) [50,51].

The various environmental labelling schemes—such as Eco-Score, Planet-Score^®^, Carbon Trust Label, Foundation Earth Eco-Impact Label, and Enviroscore—share the objective of making the environmental impacts of food products visible and understandable through scientific methodologies and life-cycle-based assessments; each takes a specific approach to addressing information needs. For instance, Eco-Score and Planet-Score^®^ offer an integrated and multi-dimensional perspective on impact, while the Carbon Trust label focuses exclusively on the carbon component. Conversely, the Foundation Earth Eco-Impact label and Enviroscore propose classification systems that facilitate immediate and easily interpretable comparisons between products [2,25,43,44,49]. Concurrently, FOPL eco-labels function as pivotal instruments of communication and transparency, furnishing unambiguous and readily accessible information regarding the environmental impact of products, whilst concurrently orienting companies and institutions towards more sustainable practices. Indeed, the harmonization of criteria and methodologies, in conjunction with the adoption of strict measures against greenwashing, is a priority for the European Union, which intends to promote reliable and comparable communication of environmental data, thus facilitating the transition towards a more sustainable economy [49,52,53].

#### 3.2.3. Processed Food Label

The degree of food processing represents an emerging criterion in food classification, increasingly considered in the context of front-of-pack nutritional labelling policies. In the scientific literature, particular attention has been given to ultra-processed foods (UPF), which are widely prevalent in the dietary patterns of various countries. In fact, according to data collected by the 2001–2018 cycles of What We Eat in America survey 58.2% of the total daily energy intake of adults comes from ultra-processed foods [54]. Consumption appears high even among the younger segments of the population: in children under four years of age, UPFs contribute to 45% of the daily energy intake [55]. In addition, available data indicate that the consumption of ultra-processed foods varies according to socio-demographic and geographical characteristics, suggesting greater exposure among socially and economically disadvantaged groups [56].

To classify food processing degree, several systems have been developed over the years. Each system adopts a different methodological approach, dividing foods into a different number of categories., as shown in Table 2.

Among the systems that classify foods into only two categories there are FSANZ (Food Standard Australia New Zealand system) [30] and the one proposed by Botelho and his research group [57,58]. Three-groups systems include NIPH (system the National Institute of Public Health of Mexico) [59], IFPRI (International Food Policy Research Institute system) [60], Louzada [61], and Siga [62,63]. The IARC system (International Agency for Research on Cancer) [64], UNC (University of North Carolina) system [64], NOVA [65], and IPAN (Institute for Physical Activity and Nutrition) [66] categorize foods into four groups. Lastly, the IFIC (International Food Information Council) system adopts a five-group classification [67].

Notably, a substantial number of ultra-processed food classification systems have been developed in South America, with fewer systems originating from Europe, United States, and Australia. A study published in 2021 analyzing global UPF consumption found a higher concentration of research publications originating from South America, particularly Brazil, indicating a focused research effort in these countries [68]. The predominance of South American classification models may therefore be attributed to this regional research emphasis, likely driven by the notably high consumption rates of ultra-processed foods observed in the area [69].

**Table 2:** Different processing-based food classification systems.

System	Origin	Number of Categories	Category	Subcategory	Examples
**NIPH** [65]	Mexico	3	Non-processed foods	Not processed	Corn, fruits, vegetables
				Locally made traditional Mexican foods	Corn tortillas
				Traditional Mexican preparation outside the home	Tacos, vegetables pie
				Modern preparations outside the home	Pizza, milkshakes
			Industrialized traditional		Corn flour for tortillas
			Modern industrialized		Corn flakes
**IARC** [68]	Europe	4	Foods with unknown process		
			Non-processed foods, consumed raw		Corn, fresh raw vegetables
			Moderately processed foods	No further cooking	Vacuum packed potato, vegetables canned in own juice or in water
				Cooked foods, from raw or moderately processed foods	Boiled grain, frozen cooked potato
			Highly processed foods		Flakes and flour, vegetables dried in oil, potato flakes
**NOVA** 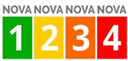 [59]	Brazil	4	Unprocessed or minimally processed foods		Grains, leafy and root vegetables, starchy roots and tubers such as potatoes, sweet potatoes, and cassava
			Processed culinary ingredients		Starches and flours
			Processed foods		Grilled vegetables in oil, canned corn with water and salt
			Ultra-processed foods		flavoured potato chips, breakfast cereals
**IFRPI** [69]	Guatemala	3	Unprocessed		Corn (staple), fruits
			Partially (Primary) processed		Corn tortillas, vegetable oils
			Highly processed.		Corn flakes, pasta products
**IFIC** [60]	USA	5	Minimally processed foods		Washed and packaged fruit and vegetables, bagged salads.
			Foods processed for preservation		Frozen fruit and vegetables, green beans
			Mixtures of combine ingredients	Packaged mixes, jarred sauce	Instant potato mix, jarred tomato sauce, tortillas
				Mixtures, home prepared	Instant potato mix, jarred tomato sauce, tortillas
			Ready-to-eat processed	Packaged ready to eat foods	Potato chips, crackers, breakfast cereal
				Mixtures, store prepared	
			Prepared foods/meals		Pies, pizzas, frozen meals
**UNC** [63]	USA	4	Unprocessed food		Fresh, frozen, or dried vegetables, brown rice
			Basic processed foods	Processed basic ingredients	whole-grain pasta, unsweetened fruit juice not from concentrate
				Processed for basic preservation or precooking	Unsweetened fruit juice from concentrate, unsweetened/unflavored canned vegetables, refined-grain pasta
			Moderately processed foods	Moderately processed for flavour	Flavoured canned, dried, refrigerated, or frozen vegetables, frozen French fries, flavoured pasta
				Moderately processed green products	Whole-grain breads/tortillas/crackers
			Highly processed foods.	Highly processed ingredients	Tomato sauce, breadcrumbs/breading with refined grains or added sugar/fat
				Highly processed stand-alone	Instant potato dishes (mashed potatoes, stuffed baked potatoes), tortillas.
**Louzada** [70]	Brazil	3	Unprocessed, minimally, or Moderately processed foods		Corn, wheat, vegetables
			Processed foods		Vegetables in brine or oil
			Ultra-processed foods		Pizzas, pies, industrialized bread
**FSANZ** [30]	Australia	2	Unprocessed		Tomato
			Processed		Canned tomato sauce
**INFOODS Botelho** [57,58]	Brazil	2	Simply Foods	Food in their natural status, being removed of non-edible or rejected parts	Fresh fruits and vegetables
				Food which one edible part has been removed during processing	Skimmed milk, white wheat flour
				Food with a single main ingredient, dehydrated or added water	Dried fruits, cooked rice
				Food with a single main ingredient, added of other ingredients in quantities that not significantly impact on energetic value	Canned tomato sauce (ingredients: tomatoes, salt)
				Food that has been processed with or without removal of edible parts with or without addition of small amount of other ingredients	Fortified corn flakes
			Composed Food		flavoured potato chips
**Siga** 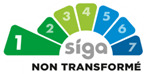 [62,71]	France	3	Un-/minimally processed	A0. Intact raw initial matrix	Wheat, fresh fruits
				A1-A2 Degraded raw metrics and culinary ingredients	Wheat flour, frozen fruits
			Processed	B1. added salt, sugars, fat ≤ official recommendations	Traditional bread, puree w/sugar fruits
				B2. added salt, sugars, fat > official recommendations	Shortbread, canned fruits
			Ultra-processed—loss of matrix/contain purified and/or denatured ingredient (excludes vitamins, minerals, tolerance of preservatives)	C01. balanced nutritional profile and one industrial ingredient/additive (acceptable)	Ravioli w/ natural flavouring, fruit drinks/ natural flavoring
				C02. high added fat/sugar/salt	Sweet crepe w/ refined oil, fruits jam w/ pectin
				C1. unprocessed industrial ingredients and/or limited additives	Soft bread w/ modified starch and aromas, fruits lollipop w/glucose syrup
**IPAN (Model 1)** [61]	Australia	4	Unprocessed or minimally processed foods		Grains, leafy and root vegetables, starchy roots and tubers such as potatoes, sweet potatoes and cassava
			Processed culinary ingredients		Starches and flours
			Processed foods	Healthy	Group 3 NOVA foods with <10 g sugar/100 g and/or <450 mg sodium/100 g (excluding cheese, which has different limits).
				Unhealthy	Group 3 NOVA foods with ≥10 g sugar/100 g and/or ≥450 mg sodium/100 g (excluding cheese, which has different limits)
			Ultra-processed foods		flavoured potato chips, breakfast cereals
**IPAN (Model 2)** [61]	Australia	4	Unprocessed or minimally processed foods		Grains, leafy and root vegetables, starchy roots and tubers such as potatoes, sweet potatoes, and cassava
			Processed culinary ingredients		Starches and flours
			Processed foods	Healthy	Group 3 NOVA foods with <10 g sugar/100 g and/or <450 mg sodium/100 g (excluding cheese, which has different limits).
				Unhealthy	Group 3 NOVA foods with ≥10 g sugar/100 g and/or ≥450 mg sodium/100 g (excluding cheese, which has different limits)
			Ultra-processed foods	Healthy	Group 4 NOVA foods with <10 g sugar/100 g and/or <450 mg sodium/100 g (excluding cheese, which has different limits).
				Unhealthy	Group 4 NOVA foods with two or more markers of ultra-processing (MUP), or with one MUP as the first ingredient, or containing flavour enhancers, or with only one MUP but exceeding sugar and/or sodium limits.

The use of different classification systems to assess the relationship between ultra-processed food (UPF) consumption and clinical outcomes can lead to conflicting results. A cross-sectional study comparing NOVA, UNC, IFIC, and IARC classifications found substantial variability in UPF intake estimates—ranging from 7.9% (NOVA) to 45.9% (IARC) of daily intake. While all systems showed a positive association with increased body weight and waist circumference, the correlations with other cardiometabolic markers were inconsistent: NOVA was linked to higher BMI, UNC to elevated blood pressure and fasting glucose, and IARC to higher HbA1c. Only NOVA did not associate UPF intake with increased total and HDL cholesterol. These findings highlight how the choice of classification system can critically influence both the quantification of UPF intake and the interpretation of its health implications, reinforcing the need for methodological consistency in nutrition research [71].

Further, other studies have compared food classification systems, highlighting both strengths and limitations. Moubarac et al., 2014, evaluated five different systems based on criteria such as specificity and clarity, ranking NOVA the highest—though this assessment was conducted by its own developers [72]. Other articles observed that estimates of UPF consumption can significantly vary a lot depending on the system used [30,72,73,74]. Additional research has examined the underlying methodologies and classification criteria, revealing significant divergences in both the conceptualization of food processing and the methodological approaches adopted [75,76].

These comparative analyses underline a key limitation: the absence of a unified framework means that studies evaluating UPF consumption and health or environmental outcomes may not be directly comparable [74]. A central challenge lies in the very definition of “processed”: while some systems incorporate health implications or specific ingredients, others argue for strictly technical criteria—such as the number and type of transformations or the addition/removal of energy and edible material [57,77]. Gibney and Forde advocate for broader analyses that account for nutrient changes, sensory properties, and additive exposure, to build more robust and meaningful systems [78]. Lastly, a recurring criticism concerns the risk of promoting unprocessed foods indiscriminately, overlooking the role of processing in improving safety, shelf life, and bioavailability [75]. Such oversimplifications may even drive unsafe choices, such as consuming raw milk [79].

Despite the proliferation of heterogenic proposals among food processing classification systems, only two systems, NOVA and SIGA, have been proposed as front-of-pack labels. NOVA classification is currently the most widespread and widely researched in the scientific literature, although it is not without criticisms. This system distinguishes foods into four groups based on the nature and intensity of the industrial processes undergone, identifying as ultra-processed foods (UPF) those subjected to multiple transformations and containing industrial ingredients that are uncommon in home cooking [65]. The NOVA classification has been extensively used to investigate the health impact of ultra-processed food (UPF) consumption. An umbrella review, including 45 meta-analyses and nearly 10 million individuals, confirmed associations between high UPF intake and increased risks of cardiovascular mortality (RR 1.50; 95% CI: 1.37–1.63; quality: very low), type 2 diabetes (dose–response RR 1.12; 95% CI:1.11–1.13; quality: moderate), and anxiety disorders (OR 1.48; 95% CI: 1.37–1.59; quality: low), among others [80]. Moreover, the consumption of ultra-processed foods is associated with a higher daily caloric intake, body weight, BMI, fat mass, and lean mass, likely due to increased extracellular water [76]. Overall, 71% of the analyses found significant associations with chronic health outcomes, highlighting the broad negative implications of UPFs [80].

The SIGA classification, developed in 2020 as an evolution of the NOVA system, represents the second food processing classification proposed for front-of-pack labelling [63]. It combines NOVA’s holistic approach with additional reductionist criteria, considering the food matrix, levels of added salt, sugar and fat, the presence of markers of ultra-processing (MUPs), and at-risk additives [75]. The system includes multiple subgroups and offers a more granular evaluation of processing intensity [81]. Despite its potential, SIGA has not yet gained the widespread adoption or recognition seen with NOVA [62,63,81].

Despite increasing interest in the role of food processing in health outcomes, no country has adopted a front-of-package labelling system based on processing levels, neither mandatorily nor voluntarily.

The degree of food processing is increasingly recognized not only for its nutritional implications but also for its environmental impact. The production systems, industrial processes, types of packaging, and patterns of excessive consumption associated with ultra-processed foods (UPFs) contribute substantially to greenhouse gas emissions, biodiversity loss, and an increased water footprint [82,83]. Evidence suggests that UPFs are generally associated with higher greenhouse gas emissions, though their relationship with water use and affordability appears less consistent. These findings underscore the importance of complementing food processing classification with additional nutritional and environmental indicators to more accurately evaluate the sustainability of dietary patterns [84].

In conclusion, NOVA is the most studied and widely used in the scientific literature, while the other food classification systems proposed have limited scientific publication to support them. Although NOVA recommendations have been endorsed by international institutions such as the WHO, significant methodological heterogeneity persists across food processing classification systems. A standardized, validated system could improve public health strategies, yet further comparative research is needed to establish a shared and scientifically grounded definition of food processing. Furthermore, including this parameter on front-of-package labels could be important for informing and protecting the consumer and for safeguarding the most vulnerable segments of the population. This requires a scientific and regulatory harmonization for a certain identification and providing consumers with clear indications on modes and quantities of consumption.

#### 3.2.4. Combined Food Label

The last decade has experienced the development of many front-of-pack labels. As already pointed out by this review, in addition to health and nutrition claims, these messages mostly vary from nutritional to food processing and environmental labels [85]. In fact, due to the increasing threat of climate change and global warming, together with the continuous developments of industrial processes on food ingredients and final food preparations, new dimensions have been added to the concept of eating patterns [21]. The health dimension of foods, previously assessed only in terms of nutrient balance, has recently been proposed to also include the aspect of food processing. As suggested by the research group that developed the Nutri-Score in a cross-sectional study, the consumption of ultra-processed foods accounts for a considerable percentage of the individuals’ overall diet quality, partially independent from the nutritional quality attribute [86]. Similarly, the prospective cohort study of Bonaccio et al., 2022, indicated that the mortality risk associated with a diet composed of nutrient-poor foods is partially explained by a high degree of food processing, whereas poor quality of foods does not explain the relation between UPFs-rich diets and mortality [26]. Concurrently, there is an increasing body of evidence on comparative analyses between Nutri-Score and NOVA classification in retailed foods in different countries [6,10]. In research conducted on the Open Food Fact database, the authors found that all Nutri-Score categories contain a substantial proportion of UPFs: 26.1%, 51.5%, 59.1%, 67.4%, and 83.7% in categories A, B, C, D, and E, respectively. They concluded the article suggesting that the Nutri-Score “should at least be accompanied by complementary labelling indicating the level of processing” [87]. 

To solve this gap, some nutrient profiling systems tried to include food processing directly into their algorithms, as in the case of Food Compass [88], even if additional examinations questioned the validity of the Food Compass Score together with the raise of some doubts on the NOVA classification usefulness [89]. Differently, the authors of Nutri-Score implemented a 2.0 label version encompassing the NOVA 4 category as an additional black-coloured ultra-processed banner surrounding the original label. Consumers showed purchase intentions directed towards the healthiest-perceived product, which was not ultra-processed in every food choice test, thus indicating the consumer’s capacity to process the dual information and a synergic effect of the two messages [1].

On the other hand, a growing body of literature is focusing on the possibility of FOPLs to encompass nutrition with environmental food information. Two studies investigated the potential of different nutrient profiling systems and FOPLs in discriminating foods according to their environmental impact [18,33]. However, in both studies, labels generally demonstrated low-to-moderate correlation values with environmental indices, indicating that these algorithms cannot be used as proxies for the sustainable dimension of eating patterns. A possible solution could be a combination of a nutritional with an ecological label, in a dual-labelling system. Most studies in this area have explored the application of the Nutri-Score together with the Eco-Score, now renamed Green-Score. De Bauw et al., 2021, evidenced that this joint association improves the nutritional quality but not the environmental quality of participants’ shopping baskets [7]. A second randomized controlled trial conducted by the same research group confirmed the inability of the dual system to raise the environmental quality of individual food choices [53]. Moreover, Jürkenbeck et al., 2024, in an online survey submitted to 1061 German consumers, revealed that the two labels influence each other in modifying consumers’ perception on food healthiness and sustainability [41]. Some foods were wrongly classified as healthier due to a low environmental score and, vice versa, environmental impacts were perceived as lower when the Nutri-Score was contemporarily displayed [41]. Nonetheless, as expected, consumers’ willingness to pay appears higher when green categories are displayed, stable with the yellow colour, and reduced in case of red ratings [90]. The same bias was also evidenced in a cross-sectional online survey, when conflicting values of Nutri-Score and Eco-Score determined a decrease in the synergic effect of the dual-system [27]. In the only study that investigated FOPLs different from the Nutri-Score, 329 young Chilean consumers were exposed to a random set of labels including the Chilean warning labels and an environmental label, either singularly or in association. Results indicated that young consumers’ food choice is mostly driven by price, followed by food environmental impact. However, consumers interested in food healthiness seem to be less interested in food sustainability and vice versa [91].

The study of Marette, 2022, provided French consumers with foods displaying Eco-Score and Nutri-Score singularly, in association, and integrated in a global score [92]. Purchase intentions were more affected by single scores than a global one, whereas, in the case of label combinations, the negative impact on purchase intentions of red ratings was higher than the positive impact of a green classification [92]. The impact of combining nutritional and environmental labels appears to be, therefore, inconclusive: only the article of Marette, 2022 [92], reported positive results, while the majority showed mixed or partial positive results, where the environmental dimension is, probably, outweighed by the nutritional aspect of foods. As already pointed out by the systematic review of Andreani et al., 2025, the limited number of studies in this area still impedes drawing any conclusion [93].

An even smaller number of publications have tried to combine or integrate all the 3 dimensions of foods: nutritional, environmental, and processing. For instance, the willingness to pay was assessed on 1250 Italian consumers to evaluate the influence of FOPLs on their purchase intentions of new food products. Results indicated that nutritional labels were the most effective information, even if FOPLs messages influence only the consumers’ perception of food but not their willingness to buy [94]. In an opinion paper published in July 2020, the authors of Nutri-Score suggested that the time to switch to a 3D-vision has come. The authors state that those “three dimensions can certainly be inter-related, but they are not collinear and correspond to complementary concepts” [24]. They propose an association of two labels, the Nutri-Score version 2.0, that flags UPFs in combination with the nutritional rating, with an organic logo that certifies environmentally friendly food production methods [24]. However, as pointed out by the study of Valenzuela et al., 2022, discrepancy between food ranking systems can interfere with consumers’ food perception as recommended or not [95]. This can lead to the application of personal coping strategies to tackle multiple information elaboration, which can result in stalled behaviours where additional messages appear counter-influential [96]. Finally, an Italian research group proposed an integrated labelling system called Mediterranean Index (Med Index); this label should help consumers to improve their adherence to the Mediterranean diet [97]. Its innovation factor relies on the inclusion of physical activity suggestions related to food caloric intakes and different flags focused on the nutritional, environmental, and social dimensions of products. In this way, the Med-Index is willing to encourage the consumption of products that respect the environment, the consumers, and all the players involved in the food value chain [98]. Other alternatives, such as the Positive Food label, which gives information on nutrition, social, environmental, and the food chain, still lack scientific publications.

In conclusion, the effort to include multiple aspects of healthy and sustainable diets in food labels is still in its beginning. Therefore, there is room for further research and development of new forms of communicating information to consumers, together with finding a trade-off between the number of messages displayed on packages and the validity of integrated algorithms that incorporate factors that are sometimes unrelated to each other.

## 4. Strengths and Limitations

To our knowledge, this is the first study evaluating, individually and in combination, the available literature on the three dimensions of FOP labels in relation to healthy and sustainable diets (i.e., nutrition, environmental sustainability, and food processing). In addition, the large number of studies included (*n* > 100) provide a broad perspective on this research area.

This review also acknowledges some limitations. The inclusion of four different topics prevented us from conducting a systematic review, thus leaving the research more prone to bias and with a lower level of evidence. Moreover, the nutritional dimension showed, by far, the largest body of evidence. Therefore, to maintain a balanced report of all dimensions, the literature found on this topic was subjected to greater synthesis, resulting in a less detailed description of the results.

## 5. Conclusions

This review has examined the evolution of front-of-pack labelling (FOPL) from basic nutrient panels to sophisticated schemes that also include environmental impact and processing level. Nutritional labels, such as Nutri-Score and Traffic Light labels, are developed to direct consumers towards healthier foods, but differ significantly in their algorithm design and visual clarity. Environmental schemes, such as Eco-Score and Planet-Score^®^, utilize life-cycle assessment to quantify sustainability yet lack a uniform regulatory framework. Process-based labels, and particularly those targeting UPFs, highlight significant public health concerns but have divergent classification criteria. Despite the recent emergence of dual and combined FOPLs (e.g., Nutri-Score 2.0, paired nutrition–environment labels), there is a lack of research, as well as rigorous evidence on their communicative power and real-world impact. Methodological heterogeneity and the risk of overloading consumers with information threaten their practical utility. To address these gaps, future studies should systematically evaluate integrated labelling formats, refine scoring algorithms for transparency, and assess behavioural outcomes across diverse consumer groups. Additionally, a bibliometric analysis defined co-occurrence clusters of key terms and co-authorship by countries. The network analyses, mapped through VOSviewer software, confirmed what emerged from the literature review, revealing a multifaceted structure in which regulatory, behavioural, health, and critical approaches coexist, but with limited attention to environmental aspects, ultra-processed foods, and combined labels. Furthermore, the co-authorship analysis identified six primary clusters among countries, predominantly industrialized and characterized by rising obesity rates (7–42.8% of adult population in OECD countries) [99]. These findings highlight the need of developing of a truly multidimensional FOPL that integrates the three aspects examined (nutritional, environmental, and processing). This objective could only be achieved by combining scientific accuracy with simplicity of presentation. It is therefore proposed to establish a global coordination of efforts in order to create a single and harmonized international labelling system, under the aegis of the WHO, possibly within the scope of the Codex Alimentarius. The proposed framework would entail the establishment of a set of unified evaluation criteria, encompassing nutritional quality, environmental impact, and the degree of processing. This framework would be supported by clear design guidelines, stakeholder training, and consumer education campaigns. It is only through this holistic, evidence-based strategy, supported by global institutions, that FOPLs can effectively guide informed, health-promoting, and sustainable food choices in diverse contexts.
